# Supplementary material for: Multilayer Nanofiber Composite Separator for Lithium-Ion Batteries with High Safety
Source: Polymers (Basel). 2019 Oct 14;11(10):1671. doi: 10.3390/polym11101671 (PMC6835787; doi:10.3390/polym11101671)
Supplement: Supplementary file 1 [file polymers-11-01671-s001.pdf]

polymers

MDPI

Article

Multilayer Nanofiber Composite Separator for Lithium-Ion Batteries with High Safety

Wenxiu Yang [1], Yanbo Liu [2,3,*], Xuemin Hu [1], Jinbo Yao [3,4], Zhijun Chen [2,4], Ming Hao [2], Wenjun Tian [2], Zheng Huang [2] and Fangying Li [2]

[1] College of Textile and Garment, Hebei University of Science and Technology, Shijiazhuang 050018, China; wenxiuyang-hbust@outlook.com (W.Y.); 13014375991@163.com (X.H.)

[2] School of Textile Science and Engineering, Wuhan Textile University, Wuhan 430200, China; 15532192903@139.com (Z.C.); 18533098646@139.com (M.H.); whf850317@126.com (W.T.); 15533605582@139.com (Z.H.); m15232132913@163.com (F.L.)

[3] School of Textiles, Tianjin Polytechnic University, Tianjin 300387, China; ywx880418@sina.com

[4] School of Chemistry and Chemical Engineering, Wuhan Textile University, Wuhan 430200, China

* Correspondence: yanboliu_wtu@sina.com (Y.L.); Tel.: +86-17743797009

Received: 28 July 2019; Accepted: 28 September 2019; Published: date

Table S1. Electrolyte uptake of separator with different materials.

	Poly ether ether ketone/ polyvinylpyrrolidone 8/82	Sb_2O_3 modified PVDF-CTFE	Aramid/ polyphenylene sulfide	poly(vinylidene fluoride-*co*-trifluoroethylene)	APEAP
Electrolyte uptake (%)	340	356	238.6	160	**592**

Electrochemical performance

Table S2. Electrochemical performance of LIBs with different separators.

Samples	Bulk resistance (Ω)	Ionic conductivity (mS cm^{-1})	EIS(Ω)	Electrochemical stability window (V)
PP/PE/PP	8.16	0.15	99	4.2
PI	0.95	1.11	78	5.0
AP	0.52	1.73	62	5.3
APEAP	0.95	2.43	69	5.2

Battery performance

Table S3. Battery performance with different separators.

Samples	Initial discharge capacities (mAh g-1)	Discharge capacity after 100 cycles (mAh g^{-1})	Capacity retention (%)
PP/PE/PP	125.6	80.7	64.3
PI	157.5	133.7	84.9
AP	169.2	146.4	86.5
APEAP	162.1	141.0	87.0
